# Development of Isothermal Recombinase Polymerase Amplification Assay for Rapid Detection of Porcine Circovirus Type 2

**DOI:** 10.1155/2017/8403642

**Published:** 2017-03-23

**Authors:** Yang Yang, Xiaodong Qin, Yingjun Sun, Guozheng Cong, Yanmin Li, Zhidong Zhang

**Affiliations:** State Key Laboratory of Veterinary Etiological Biology, Lanzhou Veterinary Research Institute, Chinese Academy of Agricultural Sciences, Xujiaping 1, Lanzhou, Gansu 730046, China

## Abstract

Porcine circovirus virus type II (PCV2) is the etiology of postweaning multisystemic wasting syndrome (PMWS), porcine dermatitis, nephropathy syndrome (PDNS), and necrotizing pneumonia. Rapid diagnosis tool for detection of PCV2 plays an important role in the disease control and eradication program. Recombinase polymerase amplification (RPA) assays using a real-time fluorescent detection (PCV2 real-time RPA assay) and RPA combined with lateral flow dipstick (PCV2 RPA LFD assay) were developed targeting the PCV2 ORF2 gene. The results showed that the sensitivity of the PCV2 real-time RPA assay was 10^2^ copies per reaction within 20 min at 37°C and the PCV2 RPA LFD assay had a detection limit of 10^2^ copies per reaction in less than 20 min at 37°C. Both assays were highly specific for PCV2, with no cross-reactions with porcine circovirus virus type 1, foot-and-mouth disease virus, pseudorabies virus, porcine parvovirus, porcine reproductive and respiratory syndrome virus, and classical swine fever virus. Therefore, the RPA assays provide a novel alternative for simple, sensitive, and specific identification of PCV2.

## 1. Introduction

Porcine circovirus virus (PCV) is a small, nonenveloped DNA virus with diameter of 17 nm, which is divided into two distinct genotypes (PCV1 and PCV2) [1]. PCV2 is a major causative agent of postweaning multisystemic wasting syndrome (PMWS) which is characterized by progressive weight loss, respiratory impairment, nephropathy syndrome, and congenital tremor, and it can affect nursery and fattening pigs all around the world [2–5]. Furthermore, as PCV2 is an immunosuppressive virus, it makes the pig more vulnerable to other viruses and bacteria. Investigations show that PCV2 commonly exists in pig populations [6].

Polymerase chain reaction- (PCR-) based assays including conventional PCR and real-time PCR assays were developed and widely used in the laboratory for detection of PCV2 DNA [7–12]. These PCR-based assays have played a crucial role in monitoring and controlling PCV2. However, these assays require sophisticated equipment and well-trained technician, which limit the application of PCR-based methods in field and poorly equipped technicians.

An isothermal detection method, recombinase polymerase amplification (RPA), has been developed as an alternative to PCR assay which can amplify nucleic acids at 37°C–39°C within 20 min. RPA has been developed for veterinary importance viruses detection, including porcine parvovirus virus [13], avian influenza virus [14], bovine viral diarrhea virus [15], canine parvovirus type 2 [16], foot-and-mouth disease virus [17], and Orf virus [18, 19]. In this study, we developed fluorescent probe-based real-time RPA assay (PCV2 real-time RPA assay) and RPA assay united with LFD (PCV2 RPA LFD assay) to detect PCV2.

## 2. Materials and Methods

### 2.1. Virus and Clinical Specimens

All viruses used in this study are listed in Table 2. Sixty-five clinical samples (spleen, inguinal lymph node, tonsil, lung, and serum) were collected from suspected PCV2 infection pigs from eight pig farms in Shandong Province (China), thirty-seven clinical samples (inguinal lymph node, tonsil, lung, and serum) were collected from Gansu Province (China), and ten PCV1 positive samples were conserved in our laboratory, confirmed by real-time qPCR [20] and Sanger dideoxy sequencing.

### 2.2. Generation of DNA Standard and DNA Extraction

The PCV2 ORF2 gene segments (320 bp, ranging from 359 bp to 678 bp of DQ231511) named pPCV2/RPA were synthesized by Genewiz (Suzhou, China) as DNA standard. Viral RNA/DNA was isolated by a TaKaRa Viral Mini kit (TaKaRa, China) according to the manufacturer's instructions and eluted in a volume of 50 *μ*L. In order to use the RPA assay in the field, DNA extraction was also performed by innuPREP MP basic kit A (Jena Analytik, Jena, Germany) combined with magnetic bead separation rack following the manufacturer's instructions.

### 2.3. Design of the Primers and Probe of RPA

After aligning the ORF2 gene consensus sequence of KR559725.1, KR559695.1, KM455975.1, KP768481.1, JN181905.1, JN181902.1, KF850461.1, KC620515.1, KF035059.1, KF951567.1, KF951570.1, KM624031.1, and JQ002672.1, we designed PCV2-specific RPA primers and probes in accordance with TwistDx RPA kits guidelines (Cambridge, United Kingdom). The sequences of designed probe and primers were summarized in Table 1.

### 2.4. Real-Time qPCR Assay

Real-time qPCR assay for detection of PCV2 DNA and PCV1 DNA were both performed on Agilent Mx3005P thermocycler machine (Life Technologies, USA) as previously described [20, 21]. The primers PCV2 F (5′-ATGGCGGGAGGAGTAGTTT-3′) and PCV2 R (5′-CCCTTTGAATACTACAGCG-3′) were used to amplify a 171 bp fragment within ORF2 region of the PCV2 genome. The detection limit was 80 copies per reaction, and it was specific for the PCV2. The primers for PCV1 DNA were as follows: PCV1 F (5′-GTCAGTGAAAATGCCAAGCAA-3′) and PCV1 R (5′-CCAAACCTTCCTCTCCGCA-3′). The length of amplified products was 152 bp. The detection limit was 100 copies per reaction, and it was specific for PCV1.

### 2.5. Recombinase Polymerase Amplification

Probe-based PCV2 real-time RPA assay was performed with TwistAmp exo kit (TwistDx, Cambridge, United Kingdom) on Agilent Technologies Mx3005P thermocycler (Life technologies, USA). The PCV2 real-time RPA assay was carried out as described below: 4x rehydration buffer, 120 nM real-time RPA probe, 420 nM real-time RPA primes, 2 *μ*L DNA template, and 14 mM magnesium acetate. Fluorescence intensity of FAM was measured once every 20 seconds. PCV2 RPA LFD assay was performed with TwistAmp nfo kit (TwistDx, Cambridge, United Kingdom) combined with lateral flow dipstick (Milenia Biotec GmbH, Germany) in a water bath at 37°C. After 20 min incubation, 2 *μ*L of amplification product was diluted in 198 *μ*L of the assay buffer.

### 2.6. Sensitivity and Specificity of PCV2 Real-Time RPA and RPA LFD Assays

A dilution range from 10^6^ to 10^1^ copies per reaction of the recombinant plasmid pPCV2/RPA DNA was used to evaluate the dynamic range of PCV2 real-time RPA assay in eight replicates. The detection limit of PCV2 RPA LFD assay was tested by a dilution series of pPCV2/RPA DNA, and the amplicons were also evaluated by agarose gel electrophoresis. In evaluating specificity of the developed PCV2 real-time RPA assay and RPA LFD assay, the reactions were evaluated with PCV2/NX, PCV2/CQ, PCV1/JL, PRRSV/CH-1R, CSFV/c-strain, PRV/Fa, PPV/AV30, and FMDV/O/CHA in three replicates.

## 3. Results

### 3.1. Sensitivity and Specificity of PCV2 Real-Time RPA Assay

**Figure 1:**
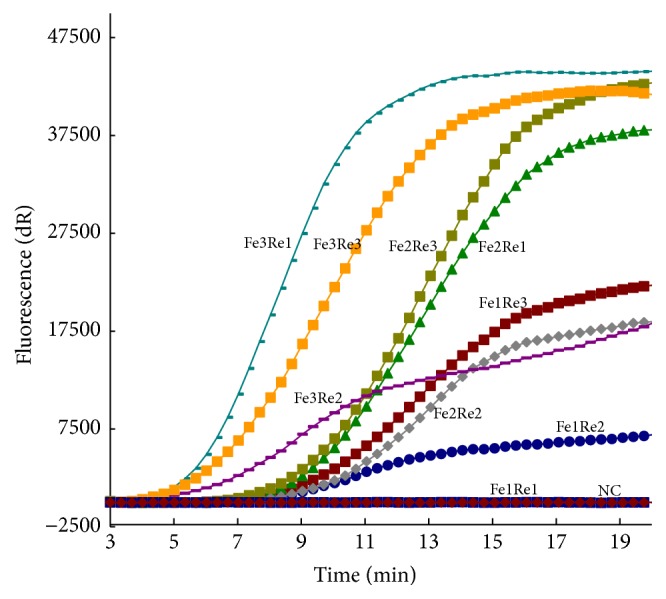
Optimal primers and probe combinations of PCV2 real-time RPA assay. The amplification results of nine different combinations of primers with the probe PCV2 RPA Pe are shown.

**Figure 2:**
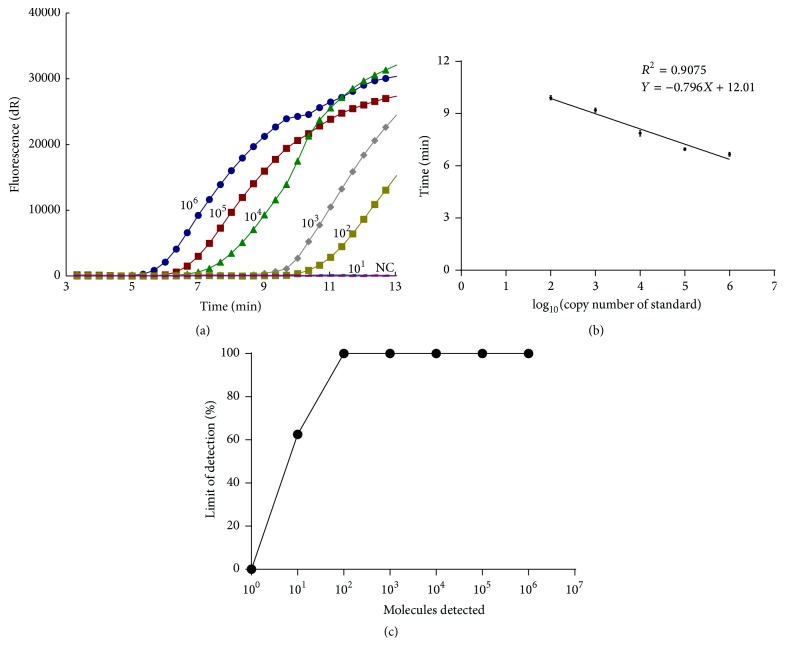
The sensitivity of PCV2 real-time RPA assay. (a) Fluorescence performance via real-time test using a dilution range of pPCV2/RPA. (b) Reproducibility of the PCV2 real-time RPA assay according to eight test runs. (c) Probit regression analysis was done on data from the eight runs of PCV2 real-time RPA assay.

After running the PCV2 real-time RPA assay with different primer and probe combinations, PCV2 RPA F3/PCV2 RPA R1 combines with PCV2 RPA Pe which were used as they yielded the highest amplification efficiency (Table 1, Figure 1). The PCV2 real-time RPA assay was sufficiently sensitive for detecting 10^2^ copies per reaction within 20 min at 37°C (Figures 2(a) and 2(b)). The limit of PCV2 real-time RPA assay detection in 95% was 10^2^ copies per reaction of pPCV2/RPA (Figure 2(c)). In evaluating the specificity of PCV2 real-time RPA assay, no cross-reactions were observed with PCV1/JL, PRRSV/CH-1R, CSFV/c-strain, PRV/Fa, PPV/AV30, and FMDV/O/CHA and positive signal was only observed with PCV2/NX and PCV2/CQ strain (Table 2).

### 3.2. Sensitivity and Specificity of PCV2 RPA LFD Assay

The sensitivity results showed that the detection limit of PCV2 RPA LFD assay was 10^2^ copies per reaction of the recombinant plasmid pPCV2/RPA DNA (Figure 4(a)), and the amplicon in the PCV2 RPA LFD assay (146 bp) was also tested by subsequent visualization with 3% agarose gel electrophoresis (Figure 4(b)). In evaluating the specificity of PCV2 RPA LFD assay, no cross-reactions were observed with other important viruses of pigs as described above (Figure 5(a)), which were also confirmed by the agarose gel electrophoresis (Figure 5(b)).

### 3.3. Performance of PCV2 Real-Time RPA Assay and PCV2 RPA LFD Assay on Clinical Samples

The practicality of both PCV2 real-time RPA assay and PCV2 RPA LFD assay was evaluated with clinical tissue samples (*n* = 102). The results were then compared with PCV2 real-time qPCR assay. Among these clinical samples, 31 samples were tested as positive by PCV2 real-time qPCR assay (CT value ranging from 19 to 30). All of these 31 samples were determined to be positive by both PCV2 real-time RPA assay (threshold time ranging from 5.6 to 9.3) and PCV2 RPA LFD assay. Based on total samples examined, the sensitivity and the specificity of both PCV2 real-time RPA assay and PCV2 RPA LFD assay for identification of PCV2 were 100% and 100%, respectively, when compared to PCV2 real-time qPCR (Table 3). The PCV2 real-time RPA assay and PCV2 RPA LFD assay were also evaluated with PCV1 positive samples (*n* = 10), and the results showed that all the samples were PCV2 negative. The extraction effects of innuPREP MP basic kit were tested with these PCV2 positive tissue samples, and results showed this kit performed well (Table 4). This DNA/RNA extraction kit combining with RPA assay could be used in the field or in not well-equipped laboratories.

## 4. Discussion

**Figure 3:**
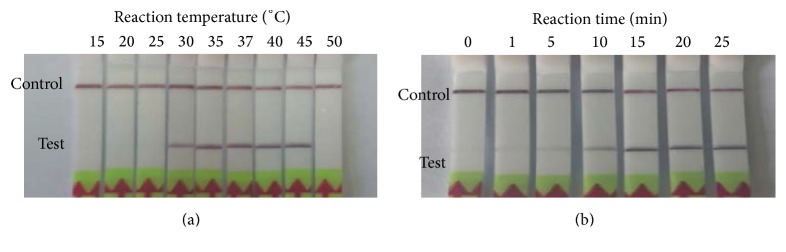
Optimal detection conditions of PCV2 RPA LFD assay. (a)The assay works in a broad range of temperatures. (b) After 10 min of amplification, the test line is visible on the lateral flow dipstick.

**Figure 4:**
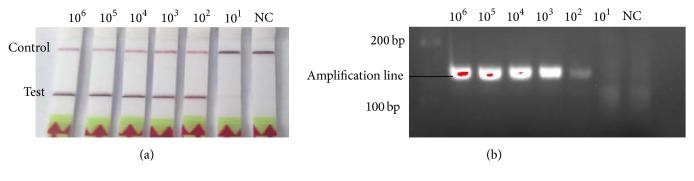
Evaluation of the sensitivity of PCV2 RPA LFD assay. (a) In the lateral flow format (PCV2 RPA LFD) the sensitivity was 10^2^ copies of the standard DNA. (b) Positive PCV2 RPA LFD reaction products (146 bp) could be detected on a stained agarose gel (3%).

**Figure 5:**
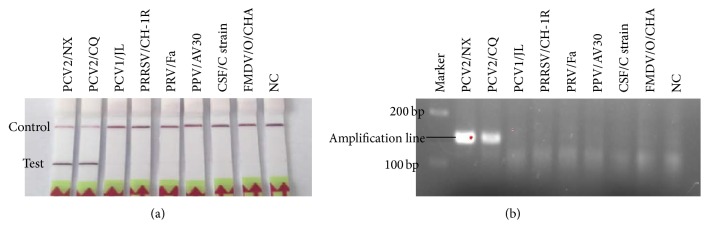
(a) Specificity test results of PCV2 RPA LFD assay using total DNA extracted from PCV2 virus and other virus. (b) Positive PCV2 RPA LFD reaction products (146 bp) could be detected on a stained agarose gel (3%).

In this study, RPA assays were developed for rapid and specific detection of PCV2. The most optimal detection conditions of PCV2 RPA LFD assay were explored with different reaction temperature and time. The results showed that it amplified well from 30°C to 45°C. The amplification products could be detected when the reaction time was more than 10 min (Figures 3(a) and 3(b)). Therefore, the PCV2 RPA LFD assay performed in a simple water bath at 37°C for 20 min arbitrarily. A visible band on the LFD strip gives a clear positive/negative result which can be easily read by the naked eyes without any training. In the developed PCV2 real-time RPA assay, a real-time PCR machine is available in our laboratory. A simple ESEQuant TS2 device (Qiagen, Germany) is also available in PCV2 real-time RPA assay [14, 17]. This device is simpler and cheaper than the real-time PCR machine, and it could be used conveniently in field as it could be charged by battery. Additionally, a commercially magnetic bead-based DNA/RNA extraction kit could combine with RPA assay without any instrument. These features made PRA more applicable for onsite testing or rapid diagnosis in not well-equipped laboratories.

Different isothermal molecule amplification assays have been developed as a rapid, simple, and cost-effective alternative to PCR-based molecule assay since 1990s, which include loop-mediated isothermal amplification assay (LAMP) [22], helicase-dependent amplification assay [23], rolling circle amplification assay [24], nucleic acid sequence-based amplification assay [25], transcription-mediated amplification assay [26], signal-mediated amplification assay [27], self-sustained sequence replication assay [28], ramification amplification assay [29], and RPA assay [30]. Among these assays, RPA has several clear advantages including no initial heating for DNA denaturation, the availability of a simple battery-charged ESEQuant TS2 instrument, and much more easiness in implementing test conditions (37°C within 20 min).

In comparison with LAMP assay for detection of PCV2 [31–34], which requires longer time (60 min) and higher temperature (62–65°C), RPA assay takes over less than 20 min at 37°C to complete, which makes RPA much simpler and easier to use. The potential defect of PCV2 RPA LFD assay is that it may carryover contamination in the field as it needs to open the tube after amplification. Therefore, precautions should be taken such as careful opening and closing of reaction tubes, frequent changing the gloves, and separating the progress of pre- and post-RPA amplification. Furthermore, replacement of dTTP with dUTP may help prevent such carryover contamination as demonstrated in PCR and LAMP assays [35, 36]. Our results showed that the developed RPA assay could tolerate base mismatch in some degree as the primes could not completely match with the two PCV2 strains used in our work based on sequence alignment, and it may detect PCV-2 strains in some degree of variability. Wang et al. developed a basic RPA assay for PCV2 detection, recently [37]. The sensitivity and specificity were almost the same as our research, but the results were analyzed using agarose gel electrophoresis and staining limiting the use of it in the field.

## 5. Conclusions

In summary, the PCV2 real-time RPA assay and PCV2 RPA LFD assay described are sensitive and specific for rapid detecting of PCV2 within less than 20 min. The developed RPA assay has the advantage of providing a potential diagnostic tool suitable for routine diagnostic use in a laboratory without complex equipment or in the field, which would greatly assist in epidemiological investigation of PCV2.

## Figures and Tables

**Table 1:** RPA primers and probes designed in this research.

Name	Sequence (5′ –3′)	Genome location (DQ231511)
PCV2 RPA Fe1	ATACCATAACCCAGCCCTTCTCCTACCACTCCCGC	443–477
PCV2 RPA Fe2	ATAACCCAGCCCTTCTCCTACCACTCCCGCTACTT	448–482
PCV2 RPA Fe3	AAACCTGTCCTAGATTCCACTATTGATTACTTCCA	490–524
PCV2 RPA Re1	TTGTATTCCTGGTCGTATATACTGTTTTCGAACGC	601–635
PCV2 RPA Re2	ATATTGTATTCCTGGTCGTATATACTGTTTTCGAA	604–638
PCV2 RPA Re3	TTACACGGATATTGTATTCCTGGTCGTATATACTG	612–646
PCV2 RPA Pe	ATTACTTCCAACCAAACAACAAAAGAAATCAGCTG	515–569
	(FAM-dT)G(THF)C(BHQ1-dT)GAGACTACAAACTGC-C3 space	
PCV2 RPA Fn1	ATACCATAACCCAGCCCTTCTCCTACCACTCCCGC	443–477
PCV2 RPA Fn2	ATAACCCAGCCCTTCTCCTACCACTCCCGCTACTT	448–482
PCV2 RPA Fn3	AAACCTGTCCTAGATTCCACTATTGATTACTTCCA	490–524
PCV2 RPA Rn1	biotin-TTGTATTCCTGGTCGTATATACTGTTTTCGAACGC	601–635
PCV2 RPA Rn2	biotin-ATATTGTATTCCTGGTCGTATATACTGTTTTCGAA	604–638
PCV2 RPA Rn3	biotin-TTACACGGATATTGTATTCCTGGTCGTATATACTG	612–646
PCV2 RPA Pn	FAM-TACTTCCAACCAAACAACAAAAGAAATCAGCTGTG	517–567
	-THF-CTGAGACTACAAACT-C3 space	

**Table 2:** Evaluation of the specificity of PCV2 real-time RPA assay and PCV2 RPA LFD assay.

Virus family	Virus species	Virus strain	Real-time RPA	RPA LFD	Real-time qPCR
Circoviridae	PCV2	NX strain	6 min	+	15 (CT)
	PCV2	CQ strain	5.6 min	+	14 (CT)
	PCV1	JL strain	−	−	−
Arteriviridae	PRRSV	CH-1R	−	−	−
Herpesviridae	PRV	Fa	−	−	−
Flaviviridae	CSF	C-strain	−	−	−
Parvoviridae	PPV	AV30	−	−	−
Picornaviridae	FMDV	FMDV/O/CHA	−	−	−

^+^Positive;  ^−^negative.

**Table 3:** Comparison of PCV2 real-time RPA assay and PCV2 RPA LFD assay with real-time qPCR assay on clinical samples.

Clinical sample	Real-time RPA		RPA LFD		Real-time qPCR	
	Positive	Negative	Positive	Negative	Positive	Negative
Spleen	4	12	4	12	4	12
Inguinal lymph node	6	19	6	19	6	19
Tonsil	9	17	9	17	9	17
Lung	7	12	7	12	7	12
Serum	5	11	5	11	5	11
Total	31	71	31	71	31	71

**Table 4:** The performance of innuPREP MP basic kit on PCV2 positive samples (*n* = 31) was tested by real-time RPA assay, RPA LFD assay, and real-time qPCR assay, respectively.

Sample name	Real-time qPCR (CT)	Real-time RPA (min)	RPA LFD
Spleen 1	25	7	+
Spleen 2	27	7	+
Spleen 3	29	7.3	+
Spleen 4	30	7.6	+
Inguinal lymph node 1	28	7	+
Inguinal lymph node 2	32	8.3	+
Inguinal lymph node 3	31	8	+
Inguinal lymph node 4	29	7.6	+
Inguinal lymph node 5	26	6.3	+
Inguinal lymph node 6	26	6.6	+
Tonsil 1	30	8	+
Tonsil 2	31	8.3	+
Tonsil 3	25	6.3	+
Tonsil 4	25	6.6	+
Tonsil 5	26	6.6	+
Tonsil 6	27	7.3	+
Tonsil 7	27	7.6	+
Tonsil 8	28	7.3	+
Tonsil 9	24	6	+
Lung 1	24	6.3	+
Lung 2	24	6.6	+
Lung 3	29	7	+
Lung 4	22	6.3	+
Lung 5	22	6.6	+
Lung 6	30	8.3	+
Lung 7	30	8.6	+
Serum 1	32	9	+
Serum 2	32	9.3	+
Serum 3	27	7.6	+
Serum 4	29	7.6	+
Serum 5	25	7.3	+
